# Machine learning constructs a diagnostic prediction model for gangrenous perforation of acute appendicitis in elderly patients

**DOI:** 10.1186/s12893-026-03753-y

**Published:** 2026-04-24

**Authors:** Jie Cheng, Yanbo Sun, Bin Yang, Yun Gong, Die Fan, Yongqilin Shuang, Yanli Li, Feng Sun

**Affiliations:** 1 Department of Gastrointestinal Surgery, The Second Affiliated Hospital of Kunming Medical University, Kunming, 650101 China; 2 Department of Urology, The Second Affiliated Hospital of Kunming Medical University, Kunming, 650101 China; 3 Department of Gynecologic Oncology, Peking University Cancer Hospital, Yunnan Cancer Hospital, The Third Affiliated Hospital of Kunming Medical University, Kunming, 650118 China; 4 Center of Hepatobiliary Gastrointestinal Cancer, Diqing Tibetan Autonomous Prefectural People’s Hospital, Shangri-La, 674499 China; 5 Department of Respiratory and Critical Care Medicine, Yan’an Hospital of Kunming City, Kunming, 650051 China

**Keywords:** Machine learning, Gangrenous perforation, Acute appendicitis, Elderly patients, Diagnosis

## Abstract

**Background:**

As life expectancy rises and elderly populations grow, acute appendicitis incidence increases, often manifesting with nonspecific symptoms that challenge diagnosis. This study applied machine learning techniques to build a predictive model for gangrenous perforation, examining clinical features and risk factors in elderly patients with acute appendicitis.

**Methods:**

We conducted a retrospective analysis of elderly patients undergoing laparoscopic appendectomy for acute appendicitis at The Second Affiliated Hospital of Kunming Medical University, China, from June 2021 to January 2024 (*n* = 251). Patients were classified into gangrenous perforation (*n* = 69) and non-gangrenous (*n* = 182) groups. For descriptive reporting, patients were randomly split into training (70%) and test (30%) sets (fixed seed); univariate comparisons and feature selection were restricted to the training set. Candidate predictors were selected by LASSO and further reduced by logistic regression. Model development and performance estimation used repeated nested cross-validation (outer 5-fold ×10 repeats; inner 5-fold; metric: AUC; seed = 123). LR_3var, XGBoost, SVM, and RF were evaluated using out-of-fold AUC, calibration, and decision curve analysis.

**Results:**

LASSO retained nine candidates; logistic regression identified three final predictors (WBC, CRP, albumin). Mean outer-fold AUCs were 0.756 ± 0.080 (LR_3var), 0.757 ± 0.078 (XGBoost), 0.757 ± 0.081 (SVM_linear), and 0.728 ± 0.083 (RF). Multivariable models outperformed single predictors; calibration and decision curves indicated acceptable calibration and potential net benefit.

**Conclusions:**

Using routine laboratory markers, multivariable models showed good discrimination for gangrenous perforation under repeated nested cross-validation. LR_3var was selected for nomogram-based risk stratification due to interpretability and practicality.

**Supplementary Information:**

The online version contains supplementary material available at 10.1186/s12893-026-03753-y.

## Introduction

Aging is advancing at an unprecedented rate all over the world as evidenced by World Health Organization that the global population of people aged 60 and above is expected to double, reaching approximately 2.1 billion by 2050, and the proportion of the population aged 60 and above will increase from 13.5% in 2020 to 22% in 2050, and this rising trend has a profound impact on the different aspects of society particularly the healthcare sector [1, 2]. The incidence rates of various geriatric diseases like acute appendicitis in the elderly patients is particularly fluctuating with a significant upward trend of 3.2% has been reported annually [3]. These vigorous rising facts pretend the higher risk of acute abdominal diseases in the elderly population.

With the rise in acute appendicitis in the elderly the condition also differs from young patients due to unique pathophysiological characteristics. A major concern in clinical treatment is high incidence of gangrene and perforation (30%-40%) which is 2–3 times higher than young patients and poses significant challenges like as high risk of postoperative complications, prolonged hospital stay and hereby increased medical costs [4–6].

The poor prognosis in elderly patients with acute appendicitis is due to multiple factors like weak immune system, degenerative changes in blood vessels, and alteration in pain sensitivity. As immune response declines, the body’s defense mechanisms become less responsive to inflammation, allowing appendiceal inflammation to progress quickly and making it difficult to control effectively [7]. The degenerative changes in blood vessels affect the blood supply of the appendix resulting in local tissue ischemia and hypoxia, thereby increasing the risk of gangrene and perforation [8]. The decrease in pain sensitivity is also a factor that cannot be ignored. In addition, reduced pain sensitivity can delay symptom recognition and presentation, further increasing the risk of advanced disease in a timely manner at the initial stage of appendicitis [8]. Thus, the clinical diagnosis and treatment become challenging in elderly population making them a high-risk group in the diagnosis and treatment of acute abdominal diseases.

Early and accurate diagnosis remains difficult. The presence of comorbidities, age-related functional decline, and atypical or subtle symptoms can limit the performance of traditional diagnostic approaches [9]. The typical migratory abdominal pain, which is a hallmark symptom of acute appendicitis, however, decrease in pain sensitivity and changes in nerve conduction often leads to absence of such typical symptoms in most elderly patients [8, 10]. Signs of peritoneal irritation, which commonly indicate intra-abdominal inflammation, are also less frequently observed in older patients (reported in approximately 28%) [11]. Even loose abdominal wall muscles and slow response also results in poor diagnosis or misdiagnosis [12]. Although routine laboratory tests such as C-reactive protein (CRP) and white blood cell count (WBC) are commonly used, their diagnostic performance may be limited by variable sensitivity and specificity and by interference from concurrent conditions in older adults, making it difficult to diagnose gangrene and perforation based on single markers alone [13, 14].

Nevertheless, the imaging techniques like ultrasonography, computed tomography (CT), and magnetic resonance imaging (MRI) offers a best aid in diagnosis of acute appendicitis in the elderly patients. But intestinal gas, position of appendix, and increased adipose tissue compromise the sensitivity of non-invasive ultrasonography in elderly patients as compared to young patients (68% vs. 92%) [15–17]. While, CT offers best alternate for ambiguous diagnosis and increased the sensitivity to 89%, for elderly patients with poor renal function but the use of contrast agents has been reported to even cause the renal failure in severe cases [18]. Nonetheless, MRI being a radiation-free imaging method has shown a high accuracy in diagnosing the complex appendicitis but the cost and time and limited access hinder widespread use in the diagnosis of appendicitis hereby necessitating the use of alternatives [19].

The rapid development of medical technology has enabled the machine learning (ML) to play an increasingly important role in the clinics, and is helpful in accurate diagnosis, and formulation and optimization of patient-based treatment plans for different diseases. Evidences from breast cancer diagnosis and classification reported that ML has shown promising performance in various medical imaging modalities such as mammography, ultrasound, MRI, histology, and thermography with high diagnostic accuracy [20]. It has improved the precision of decision-making and patients’ treatment outcomes in clinics, thus offering a great potential in clinical diagnosis, personalized treatment, and health monitoring [21]. Nevertheless, the application of ML technology in diagnosis of gangrene and perforation of acute appendicitis in the elderly is still in the initial exploration stage with limited reports. Thus, in this study, we utilized the ML technology to construct a diagnostic prediction model of gangrene and perforation of acute appendicitis in the elderly patients, hereby focusing on in-depth analysis of the clinical characteristics of gangrene and perforation of acute appendicitis to provide efficient and reliable auxiliary diagnostic tools for gastrointestinal surgeons.

## Materials and methods

A retrospective study from June 2021 to January 2024 was carried out at the Second Affiliated Hospital of Kunming Medical University, China. The research protocol and consent to participate was approved by the ethical Review Committee of The Second Affiliated Hospital of Kunming Medical University, China.

### Patient selection

The inclusion criteria for patients was as: the patients were diagnosed with acute appendicitis through a comprehensive diagnosis based on pre-operative classic clinical manifestations (migratory right lower quadrant pain, tenderness and rebound tenderness at McBurney’s point), imaging features (abdominal CT showing thickening of the appendix and exudation of surrounding fat streaks), and laboratory indicators (elevated white blood cell count), and were confirmed by laparoscopic surgical resection combined with intraoperative exploration and postoperative pathological examination; having age ≥ 60 years old with complete perioperative data, including pre-operative CT imaging data, laboratory tests (complete blood count, comprehensive biochemistry, coagulation function, electrolytes), and postoperative pathological reports.

The patients with following complications like active infection in other parts, history of malignant tumor or postoperative pathological examination suggesting a neoplastic lesion, abnormal coagulation function or use of anticoagulant/antiplatelet drugs within 30 days before surgery, hematological diseases, and severe hepatic and renal insufficiency were not included in the study.

### Study parameters

The data collected was categorized into baseline data, per-operative laboratory indicators, and imaging features and pathological and intraoperative records. Briefly the baseline data comprised of demographic characteristics (age, gender, weight, height, BMI), underlying diseases (diabetes, hypertension), lifestyle habits (smoking, drinking), symptomatic characteristics (migratory right lower quadrant pain, nausea and vomiting, anorexia, fever, tenderness and rebound tenderness in the right lower quadrant), and disease course-related indicators (time from onset to surgery, use of preoperative antibacterial drugs). Thepre-operative laboratory indicators included inflammatory markers (white blood cell count WBC, C-reactive protein CRP, procalcitonin PCT), hematological parameters (platelet PLT, hemoglobin HGB, hematocrit HCT, red blood cell distribution width RDW), metabolic indicators (albumin Alb, serum Na+, direct/indirect bilirubin DBIL/IBIL), and coagulation function (D-D dimer), and the pre-operative imaging features were the minimum diameter of the appendix, thickening of the appendiceal wall (defined as thickening when > 3 mm), the presence of fecaliths in the appendiceal lumen, and the presence of surrounding fluid. While, the pathological and intraoperative records withhold verifying the accuracy of the clinical diagnosis based on the postoperative pathological diagnosis and surgical exploration results. The data were extracted through a structured electronic medical record system to ensure the integrity and consistency of the information, providing comprehensive data support for subsequent multivariate analysis.

### Data processing, feature selection, and model development

After data extraction, records were checked for plausibility and internal consistency, and entries with obvious logical errors were excluded. Because complete perioperative data were required by the inclusion criteria, a complete-case analytic dataset was used; therefore, no missing-data imputation was performed.

For descriptive reporting, the dataset was randomly split into a training set (70%) and a test set (30%) using a fixed random seed (7:3 ratio). This split was used for descriptive reporting only.

Feature selection was conducted in the training set only. LASSO regression was applied to identify candidate predictors while reducing dimensionality and mitigating multicollinearity [22]. Candidate predictors retained by the λ1se criterion were subsequently examined using univariable and multivariable logistic regression in the training set, and predictors remaining independently associated with the outcome were retained as the final predictor set for model development [22]. The final predictor set was fixed for subsequent modeling; no further feature selection was performed during cross-validation [23].

Model development and performance evaluation were primarily based on repeated nested cross-validation to reduce optimism from a single random split and to avoid overfitting during hyperparameter tuning [24, 25]. Specifically, an outer 5-fold cross-validation was repeated 10 times, and an inner 5-fold cross-validation was used for hyperparameter optimization (evaluation metric: AUC; random seed: 123). Within each outer fold, any preprocessing required for model fitting (e.g., feature scaling for SVM) and all hyperparameter tuning were performed exclusively within the corresponding training partition, and performance was evaluated on the held-out partition [26]. Out-of-fold predictions from the outer folds were used for discrimination, calibration, and decision-curve analyses [27, 28]. This design ensured that the held-out data in each outer fold were not used in any preprocessing, feature selection, or hyperparameter optimization steps [29].

The prediction models included a 3-variable logistic regression model (LR_3var), XGBoost, SVM, and RF [30, 31]. Hyperparameters were optimized within the inner cross-validation loop. For XGBoost, candidate hyperparameter combinations were sampled from a prespecified grid within each inner loop, and the number of boosting rounds was selected using early stopping. For RF, the number of trees, mtry, and node size were tuned by random search within prespecified ranges [25]. For SVM, kernel functions (linear, polynomial, radial, and sigmoid) were compared, and the cost parameter was tuned within the inner loop for each kernel; the final SVM used a linear kernel with *C* = 10. LR_3var was fitted as a standard multivariable logistic regression model without additional hyperparameters. In addition to AUC, accuracy, sensitivity, specificity, and Brier score were summarized [27].

### Statistical analysis

This study systematically analyzed 38 clinical characteristics of 251 patients based on the R statistical platform (version number 4.4.2; access website: https://www.R-project.org). In the data preprocessing stage, firstly, the Kolmogorov-Smirnov test was used to evaluate the normal distribution characteristics of continuous variables: for the indicators that conform to the normal distribution, the independent samples T-test was adopted, and the results were presented as mean ± standard deviation [$\overline{X}\pm{S}$]. For the data with non-normal distribution, the Mann-Whitney rank sum test was used, and it was described by the median and interquartile range (first quartile, third quartile) [M(*P* 25, *P* 75)]. Categorical variables were analyzed by the chi-square test, and the frequency distribution was expressed as the number of cases and the proportion [*N*(%)]. All hypothesis testing for group comparisons was performed in the training set only, and a two-sided *P* value < 0.05 was considered statistically significant. The R packages used were: ‘caret’, ‘tidyverse’, ‘autoReg’, ‘ggplot2’, ‘compareGroups’, ‘Table 1’, ‘plyr’, ‘corrplot’, ‘glmnet’, ‘rrtable’, ‘Hmisc’,‘reportROC’, ‘rmda’, ‘randomForest’, ‘dplyr’, ‘rms’, ‘data.table’, ‘xgboost’, ‘ggpubr’, ‘Matrix’, ‘e1071’, ‘nortest’, ‘plotly’, ‘Ckmeans.1d.dp’, ‘ggprism’, ‘CBCgrps’, ‘DiagrammeR’,‘shapviz’, and ‘pROC’.

**Table 1:** Training-set baseline characteristics

Characteristics	Training Set (*n* = 177)		
	Non-gangrene and Perforation	Gangrene and Perforation	*P*-value
Patients ( *n* )	128	49	
Age (years) (IQR)	68 (64,73.25)	68 (64,75)	0.654
Weight (kg)	60.47 ± 8.97	57.55 ± 9.78	0.060
Height (cm)	161.09 ± 7.65	160.76 ± 7.01	0.788
BMI (kg/m^2^)	23.32 ± 3.25	22.2 ± 3.03	0.034
Gender (*n*, %)			
Female	72(56)	22(45)	0.236
Male	56(44)	27(55)	
Diabetes (*n*, %)			
No	115(90)	45(92)	0.783
Yes	13(10)	4(8)	
Hypertension (*n*, %)			
No	80 (62)	35 (71)	0.348
Yes	48 (38)	14 (29)	
Smoking (*n*, %)			
No	117(91)	37(76)	0.01
Yes	11(9)	12(24)	
Drinking (*n*, %)			
No	120(94)	45(92)	0.74
Yes	8(6)	4(8)	
Time from Onset to Surgery (d) (IQR)			
	1(1,2)	1(1,3)	0.12
Use of Antibacterial Drugs (*n*, %)			
No	70(55)	23(47)	0.45
Yes	58(45)	26(53)	
Migratory Right Lower Quadrant Pain (*n*, %)			
Yes	128(100)	49(100)	1
Nausea and Vomiting (*n*, %)			
No	64(50)	28(57)	0.495
Yes	64(50)	21(43)	
Anorexia (*n*, %)			
No	28(22)	7(14)	0.356
Yes	100(78)	42(86)	
Fever (*n*, %)			
No	114(89)	32(65)	< 0.001
Yes	14(11)	17(35)	
Tenderness in Right Lower Quadrant (*n*, %)			
No	7(5)	1(2)	0.447
Yes	121(95)	48(98)	
Rebound Tenderness in Right Lower Quadrant (*n*, %)			
No	33(26)	12(24)	1
Yes	95(74)	37(76)	

## Results

### General clinical characteristics

A total of 251 elderly patients who underwent laparoscopic appendectomy were included (gangrenous perforation, *n* = 69; non-gangrenous, *n* = 182). The cohort was randomly divided into a training set (*n* = 177; gangrenous perforation, *n* = 49) and a test set (*n* = 74; gangrenous perforation, *n* = 20). Baseline characteristics in the training set are summarized in Table 1. Univariate comparisons were performed in the training set only. Significant between-group differences were observed in BMI (*P* = 0.034), smoking history (*P* = 0.010), and fever history (*P* < 0.001) (Table 1).

### Preoperative laboratory tests

Preoperative laboratory indices in the training set are presented in Table 2. The gangrenous perforation group differed from the non-gangrenous group in WBC (*P* = 0.001), neutrophils (*P* = 0.004), lymphocyte percentage (*P* < 0.001), CRP (*P* < 0.001), procalcitonin (*P* < 0.001), D-dimer (*P* < 0.001), indirect bilirubin (*P* = 0.030), albumin (*P* < 0.001), and serum sodium (*P* = 0.012) (Table 2).

**Table 2:** Training-set laboratory indices

Characteristics	Training Set (*n* = 177)		
	Non-gangrene and Perforation	Gangrene and Perforation	*P*-value
Patients ( *n* )	128	49	
PLT (*10^9/L)	217.16 ± 65.51	203.33 ± 54.16	0.155
WBC (*10^9/L)	11.18 ± 3.85	13.64 ± 4.56	0.001
NEUT (%)(IQR)	83.65 (73.65,88.73)	87(82.6,91)	0.004
LYM (%)(IQR)	11.5(7.25,19.7)	8.3(4.9,11.5)	< 0.001
HGB (g/L)	144.62 ± 14.69	146.92 ± 19.68	0.46
HCT (%)(IQR)	0.43(0.41,0.46)	0.43(0.4,0.48)	0.51
MCV (fL)	92.08 ± 4.34	92.66 ± 4.59	0.448
MCHC (g/L)(IQR)	337 (331.75,342)	339(328,347)	0.585
RDW (fL)	44.03 ± 3.02	44.27 ± 3.42	0.648
MPV (fL)(IQR)	9.9(9.17,10.7)	10(9.4,10.4)	0.887
PCT (µg/L)(IQR)	0.11(0.06,0.32)	0.84(0.19,7.62)	< 0.001
CRP (mg/L)(IQR)	42.34 (13.59,70.15)	91.39 (44.21,142.11)	< 0.001
D-Dimer (µg/L)(IQR)	0.51(0.34,0.99)	1.08(0.66,1.98)	< 0.001
DBIL(µmol/L)(IQR)	4.9(3.48,7)	5.5(3.6,8.1)	0.231
IBIL(µmol/L)(IQR)	15.25 (11.1,22.45)	19.9(12.9,27.9)	0.03
ALB(g/L)(IQR)	40.65 (38.48,42.73)	38.6(35.7,41.2)	< 0.001
Na^+^(mmol/L)	136.09 ± 3.10	132.65 ± 14.53	0.012

*Abbreviations CRP * C-reactive protein, *WBC*  white blood cell

### Preoperative imaging examinations

Preoperative imaging characteristics in the training set are shown in Table 3. Significant between-group differences were observed across the imaging features evaluated (Table 3).

**Table 3:** Training-set imaging features

Characteristics	Training Set (*n* = 177)		
	Non-gangrene and Perforation	Gangrene and Perforation	*P*-value
Patients ( *n* )	128	49	
Minimum diameter of the appendix (cm) (IQR)	0.9(0.7,1)	0.9(0.8,1.1)	**0.040**
Appendiceal wall thickening (*n*, %)			
No	18(14)	0(0)	**0.004**
Yes	110(86)	49(100)	
Fecalith in the appendiceal lumen (*n*, %)			
No	66(52)	34(69)	**0.049**
Yes	62(48)	15(31)	
Fluid around the appendix (*n*, %)			
No	28(22)	3(6)	**0.025**
Yes	100(78)	46(94)	

### Feature selection and derivation of the final predictor set

**Fig. 1:**
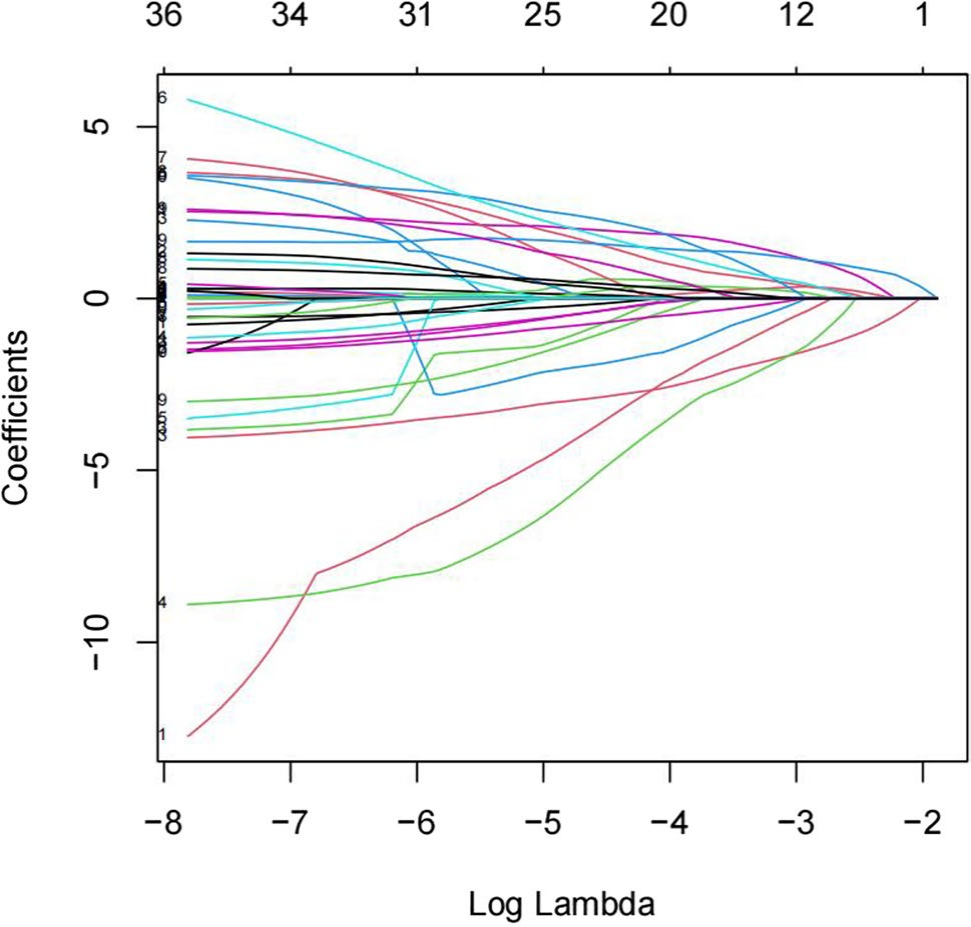
LASSO coefficient profiles across log(λ). Coefficients of candidate predictors are shown as a function of log(λ), illustrating progressive shrinkage with increasing penalization

**Fig. 2:**
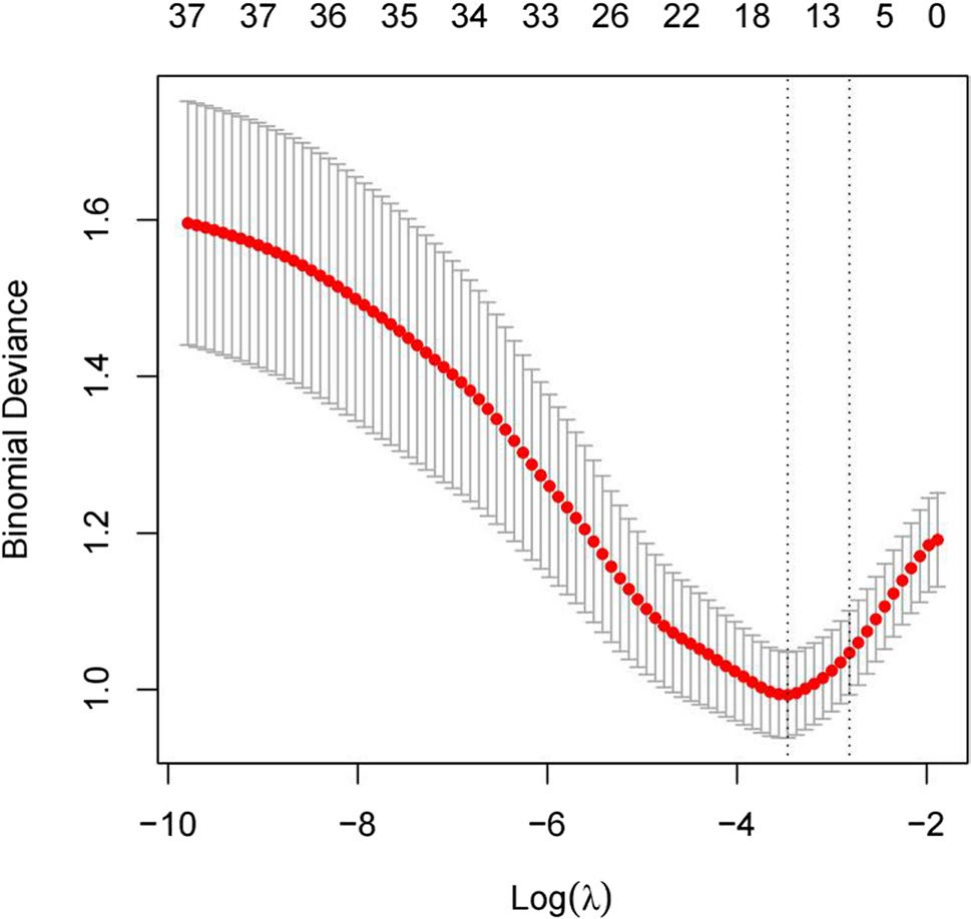
Cross-validated error for LASSO feature selection. Cross-validated error is plotted against log(λ). Vertical dashed lines indicate λmin (minimum cross-validated error) and λ1se (largest λ within one standard error of the minimum). The λ1se criterion was selected to obtain a more parsimonious candidate predictor set

Using the 177 patients in the training set, gangrenous perforation was used as the outcome for feature selection. LASSO regression was applied to reduce dimensionality and mitigate multicollinearity among candidate predictors. The coefficient trajectories across log(λ) are shown in Fig. 1, demonstrating progressive shrinkage with increasing penalization. The cross-validation error curve is shown in Fig. 2, in which λmin corresponds to the minimum cross-validated error and λ1se represents the largest λ within one standard error of that minimum. To favor a more parsimonious and potentially more robust feature set in this limited-sample setting, the λ1se criterion was selected. Accordingly, nine candidate predictors were retained for subsequent analyses: smoking history, fever history, white blood cell count, lymphocyte percentage, C-reactive protein, procalcitonin, albumin, serum sodium level, and appendiceal wall thickening.

These nine candidate predictors were then evaluated in the training set using univariable and multivariable logistic regression (Supplementary Table S1). All nine variables were significant in univariable analyses, whereas three variables remained independently associated with gangrenous perforation in the multivariable model: WBC (*OR* = 1.12, 95% CI 1.01–1.25, *P* = 0.038), CRP (*OR* = 1.01, 95% CI 1.00–1.01, *P* = 0.041), and albumin (*OR* = 0.85, 95% CI 0.76–0.95, *P* = 0.004). These three routinely available laboratory variables were retained as the final predictors for subsequent model development.

### Model development under repeated nested cross-validation

Using the final three predictors (WBC, CRP, and albumin), four models (LR_3var, XGBoost, SVM, and RF) were developed and evaluated under the repeated nested cross-validation framework described above (outer 5-fold cross-validation repeated 10 times with inner 5-fold tuning; metric: AUC; seed = 123). AUC estimates were derived from held-out predictions in the outer folds. Performance of the four multivariable models is summarized in Table 4.

**Table 4:** Performance of the four multivariable models estimated by repeated nested cross-validation

Model	Mean outer-fold AUC ± SD	Accuracy ± SD	Sensitivity ± SD	Specificity ± SD	Brier score
LR_3var	0.756 ± 0.080	0.759 ± 0.077	0.770 ± 0.130	0.731 ± 0.144	0.163
XGBoost	0.757 ± 0.078	0.754 ± 0.076	0.765 ± 0.124	0.728 ± 0.124	0.199
RF	0.728 ± 0.083	0.756 ± 0.078	0.793 ± 0.144	0.658 ± 0.185	0.173
SVM_linear	0.757 ± 0.081	0.751 ± 0.090	0.744 ± 0.123	0.753 ± 0.153	0.167

Performance metrics were calculated from out-of-fold predictions obtained in the outer loop of repeated nested cross-validation (outer 5-fold repeated 10 times; inner 5-fold for tuning; metric: AUC; seed = 123). For SVM, the reported results correspond to the selected linear kernel identified within the inner-loop kernel comparison (linear, polynomial, radial, sigmoid)

*Abbreviations AUC* area under the receiver operating characteristic curve, * CV* cross-validation, *SD* standard deviation

For SVM, kernel functions were compared during model development; the linear kernel achieved the highest mean outer-fold AUC and was selected for subsequent analyses (Supplementary Figure S1). Feature importance for the SVM_linear and a decision boundary visualization are provided in Supplementary Figures S2–S3. For XGBoost and RF, feature attribution analyses are provided in Supplementary Figures S4–S6.

### Model interpretability

To support interpretability, SHAP was used to quantify feature contributions in XGBoost. CRP showed the largest overall contribution, followed by WBC and albumin (Supplementary Figure S4), consistent with XGBoost gain-based importance (Supplementary Figure S5). In RF, feature importance showed a similar pattern, with CRP ranked highest, followed by WBC and albumin (Supplementary Figure S6). In contrast, permutation-based importance for the SVM_linear ranked WBC highest, followed by CRP and albumin (Supplementary Figure S2).

### Comparative discrimination and incremental value over single predictors

**Fig. 3:**
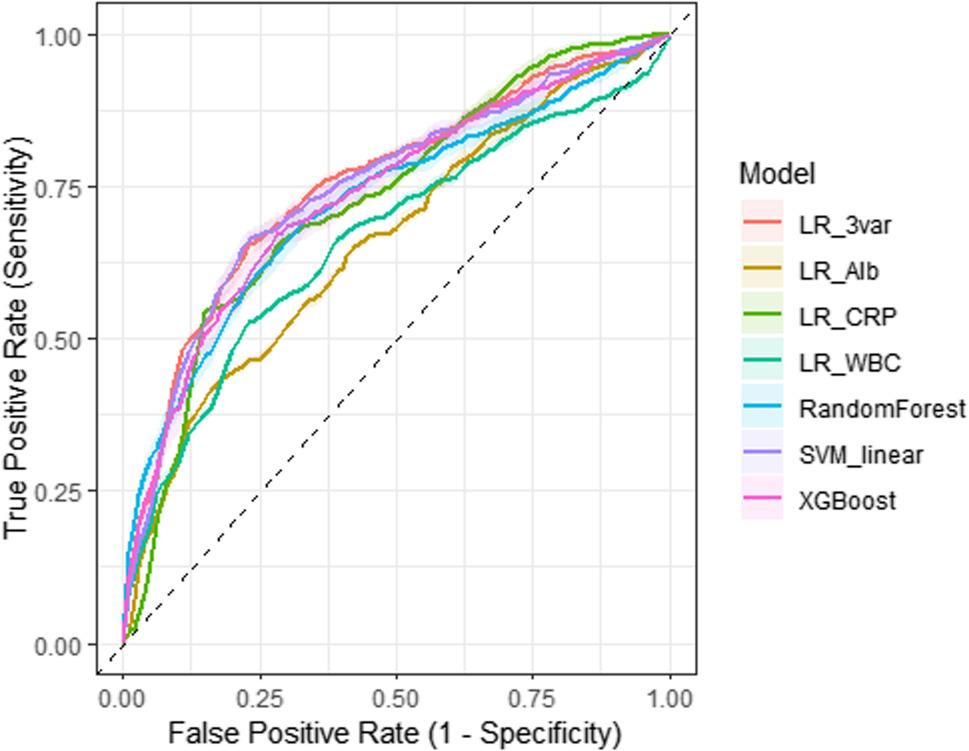
ROC curves based on out-of-fold predictions. Receiver operating characteristic curves are shown for the four multivariable models (LR_3var, XGBoost, SVM_linear, and RF) and the corresponding univariable logistic models (WBC alone, CRP alone, and albumin alone). Curves were generated using out-of-fold predictions from the outer loop of repeated nested cross-validation

To compare discrimination after feature selection, performance was evaluated using out-of-fold (OOF) predictions from repeated nested cross-validation, thereby avoiding optimistic bias from a single train–test split. ROC curves for the four multivariable models (LR_3var, XGBoost, SVM_linear, and RF) and the corresponding univariable logistic models (WBC alone, CRP alone, and albumin alone) are presented in Fig. 3. Overall, multivariable models demonstrated higher discrimination than single predictors, supporting incremental predictive value beyond any individual laboratory marker.

**Fig. 4:**
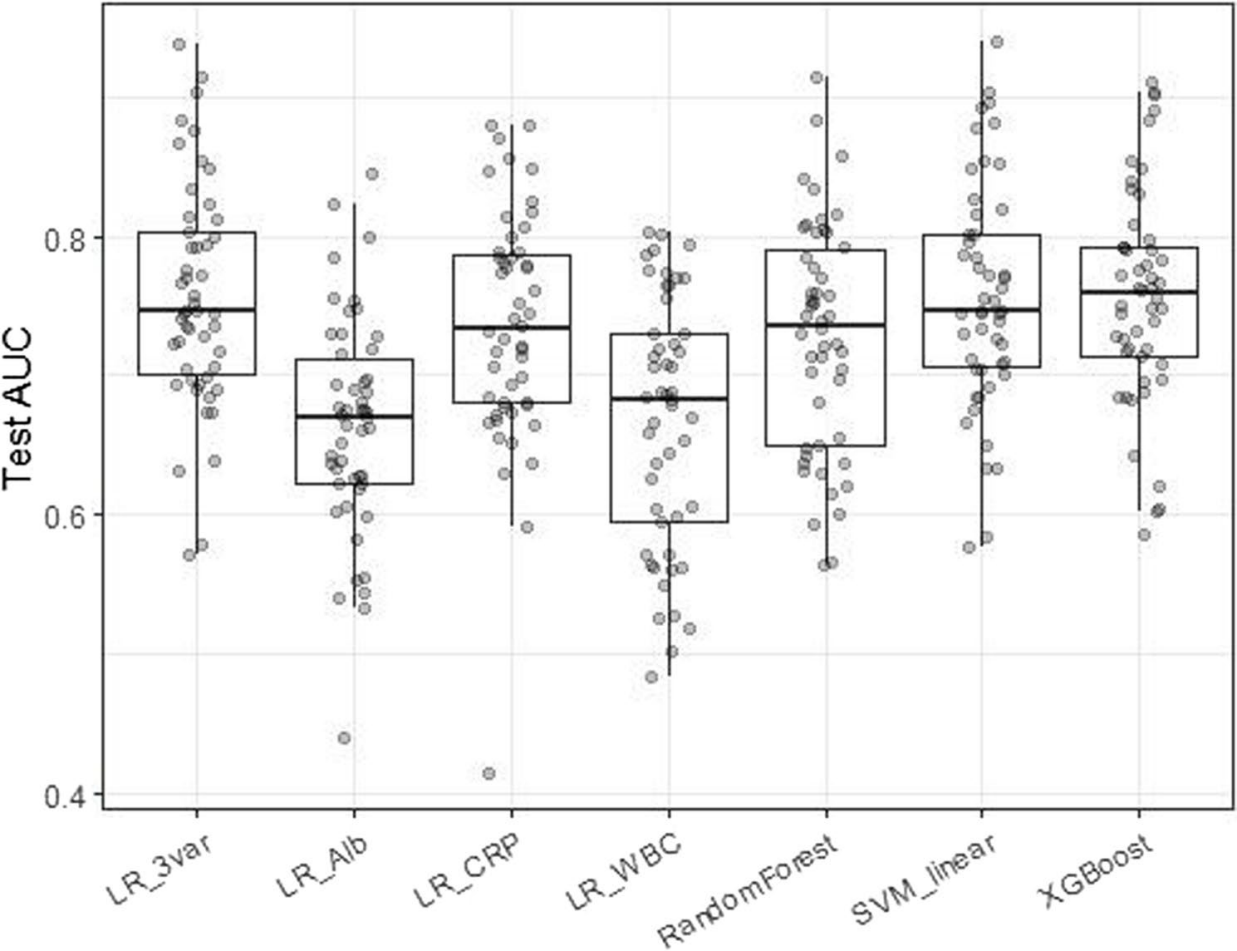
Distribution of AUC across repeated outer folds. Boxplots show the distribution of AUC values obtained from held-out outer folds across repeated nested cross-validation, reflecting performance stability under resampling

The distribution of AUC estimates across repeated outer folds is shown in Fig. 4, illustrating model stability under resampling. In paired comparisons across complete resampling blocks, overall differences in AUC were observed among the seven approaches (Friedman χ² = 104.28, *df* = 6, *P* < 2.2 × 10^− 16^; Kendall’s *W* = 0.348). Pairwise post-hoc comparisons are provided in Supplementary Table S2. Compared with univariable models, the multivariable approaches showed significantly higher AUC than WBC alone and albumin alone in post-hoc Nemenyi comparisons following the Friedman test (both *P* < 1 × 10^− 7^), and showed a modest but statistically significant improvement over CRP alone (*P* = 0.0358).

### Calibration performance based on out-of-fold predictions

**Fig. 5:**
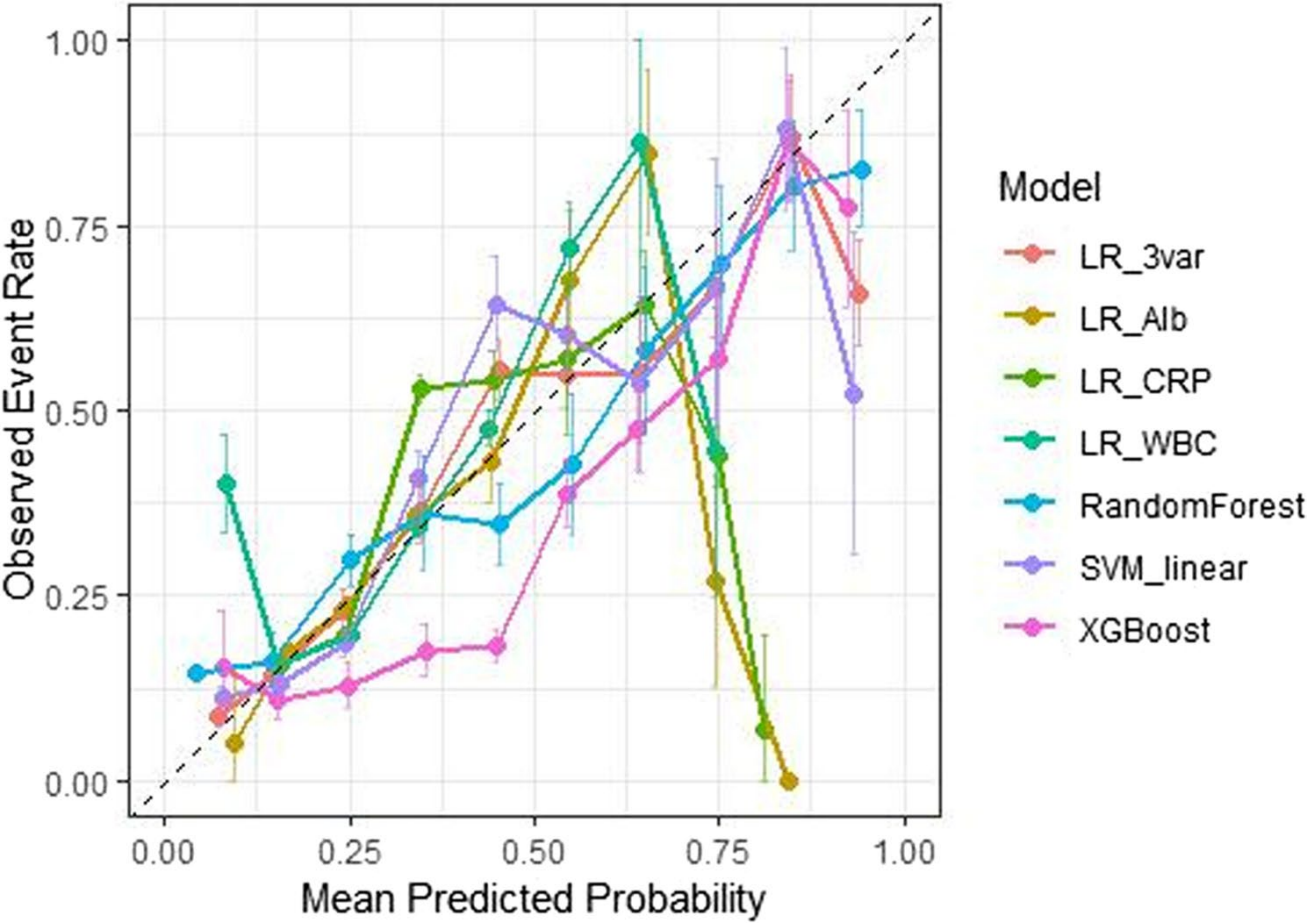
Calibration curves based on out-of-fold predictions. Calibration curves depict agreement between predicted probabilities and observed event rates using out-of-fold predictions from repeated nested cross-validation

Calibration was assessed using OOF predictions from the repeated nested cross-validation procedure. The calibration curves are shown in Fig. 5. Predicted risks were generally consistent with observed event rates across the evaluated probability range. For LR_3var, the Brier score further supported acceptable overall calibration (mean Brier score = 0.163) (Table 4).

### Decision curve analysis

**Fig. 6:**
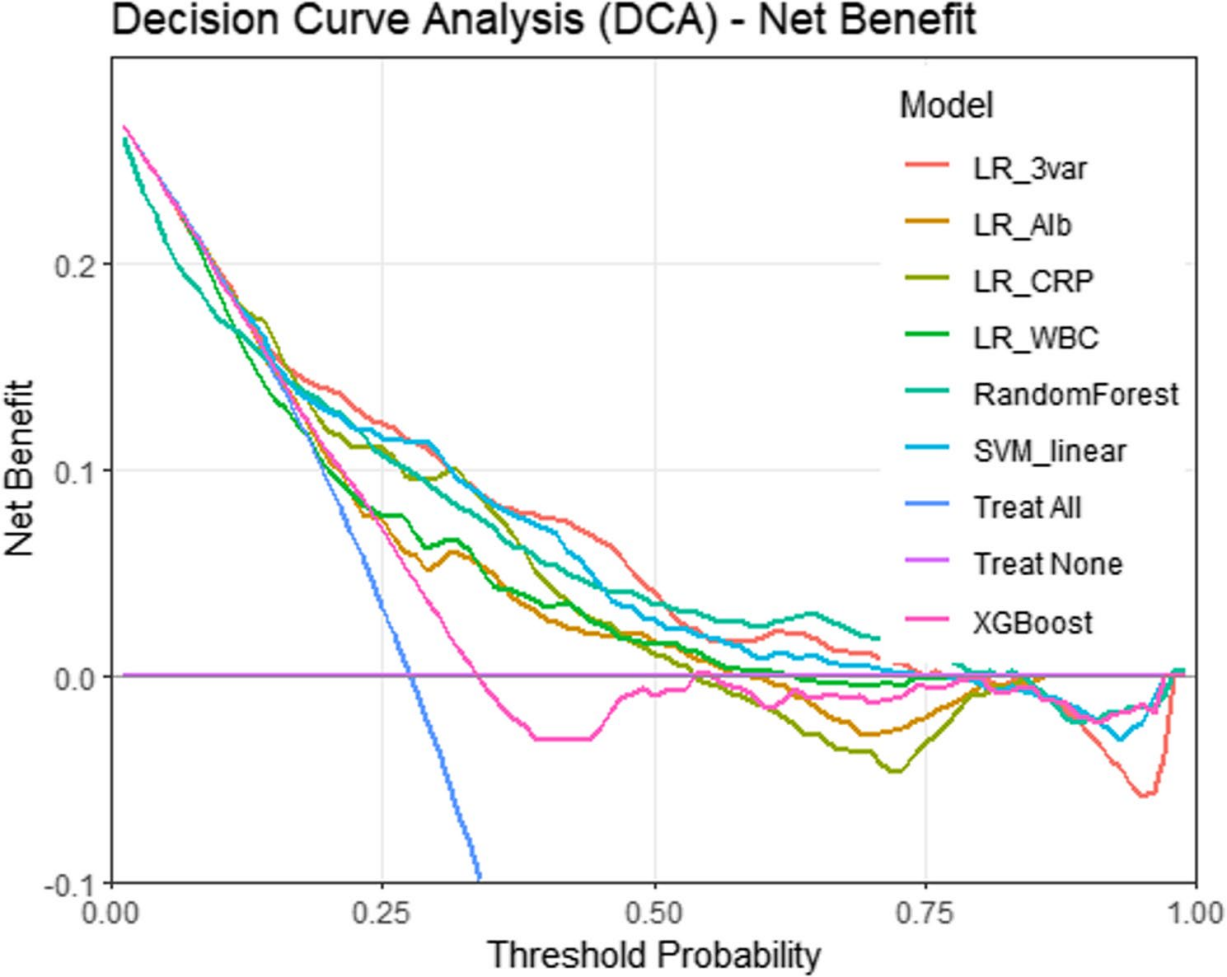
Decision curve analysis based on out-of-fold predictions. Decision curves show the net benefit of each multivariable model across a range of threshold probabilities using out-of-fold predictions, compared with “treat-all” and “treat-none” strategies

Clinical utility was evaluated by decision curve analysis using the same OOF predictions. As shown in Fig. 6, the multivariable models provided net benefit over the “treat-all” and “treat-none” strategies across clinically relevant threshold probabilities, supporting potential usefulness for assisting preoperative risk stratification in elderly patients.

### Clinical presentation of the final model (nomogram)

**Fig. 7:**
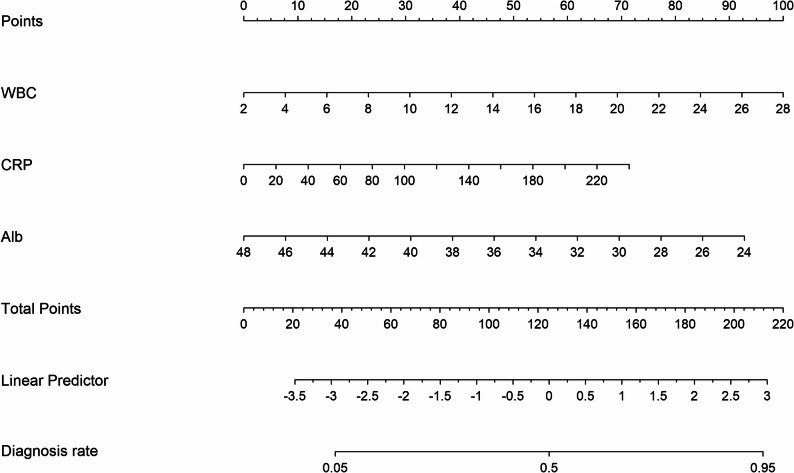
Nomogram of the final three-variable logistic regression model. A nomogram was constructed from the final three-variable logistic regression model (WBC, CRP, and albumin) refitted on the full dataset for clinical presentation

Considering discrimination, calibration, and clinical interpretability, LR_3var was selected for clinical presentation. The final LR_3var model was refitted on the full dataset using WBC, CRP, and albumin to construct a nomogram (Fig. 7). This refit was performed for clinical presentation only; performance estimates were derived from the repeated nested cross-validation framework. The regression coefficients and effect estimates of the final LR_3var model are provided in Table 5. The nomogram provides an intuitive tool for estimating the probability of gangrenous perforation by summing the point values assigned to each predictor and mapping the total score to an individualized predicted risk.

**Table 5:** Final multivariable logistic regression model with three predictors

Variable	Beta coefficient	Odds ratio (OR)	95% CI for OR	*P*-value
WBC	0.1298	1.1386	1.0521–1.2390	0.0018
CRP	0.0100	1.0101	1.0048–1.0156	0.0002
Alb	-0.1305	0.8777	0.8076–0.9490	0.0014

*Abbreviations CI* confidence interval, *WBC* white blood cell count, *CRP* C-reactive protein, *Alb* albumin

## Discussion

Acute appendicitis in the elderly tends to progress more rapidly and severely than younger individuals and this accelerated progression is attributed to the unique pathophysiology [32]. When the lumen of the appendix is obstructed, bacteria proliferate within the closed environment, triggering a severe inflammatory response which in turn impairs the blood supply to the appendix, leading to gangrene and perforation, hereby exacerbating the inflammatory cascade [33]. Clinically, this is manifested as initial periumbilical pain that migrates to the right lower quadrant, accompanied by gastrointestinal symptoms such as nausea, vomiting, and abdominal distension, as well as systemic symptoms like fever [34]. However, the disease progresses more rapidly in elderly patients due to diminished physiological functions and weakened inflammatory response. Once gangrene and perforation occur, the spread of inflammation can easily lead to severe peritonitis, extensive intra-abdominal infection, and even septic shock, severely compromising the function of multiple organ systems [35]. Given the vulnerability of elderly patients to prolonged illness, it is crucial to ensure the timely medical intervention.

The diagnosis of gangrenous perforation in elderly acute appendicitis is clinically challenging due to frequently atypical symptoms, such as mild abdominal pain, minimal gastrointestinal reactions, and less pronounced fever, which can easily be ignored by both patients and clinicians [36]. Laboratory tests including WBC count and neutrophil percentage may not show significant elevation, often reflecting the blunted inflammatory response is in this population. The malposition of appendix or intra-abdominal gas often interfere the imaging modalities such as abdominal ultrasound hereby limiting the diagnosis of appendiceal gangrene and perforation. Although CT is highly valuable for diagnosis, however, the health conditions of some elderly patients may not tolerate the procedure. Furthermore, the presence of multiple comorbidities in elderly patients can mask the symptoms of appendiceal gangrene and perforation thereby increasing the likelihood of misdiagnosis or delayed diagnosis [37].

ML has emerged as a powerful tool in the development of diagnostic models for various diseases, demonstrating the superior performance compared to traditional statistical methods [38]. Unlike conventional approaches, ML enables computers to “learn” from data without explicit programming, allowing the efficient handling of large datasets through complex interactions. By uncovering intricate patterns and associations within the data, ML provides the researchers with novel analytical perspectives and is increasingly playing a vital role in the medical research [39].

In the present study, WBC, CRP, and albumin were identified as independent predictors of gangrenous perforation in elderly patients with acute appendicitis. After LASSO-based feature selection in the training set, the candidate predictor set was refined by univariable and multivariable logistic regression, yielding a parsimonious three-variable predictor set composed of routinely available laboratory markers (Supplementary Table S1; Table 5).

Using this three-variable predictor set, four models (LR_3var, XGBoost, SVM, and RF) were developed and evaluated under a repeated nested cross-validation framework to reduce optimism from a single split and to avoid information leakage during hyperparameter tuning [29]. Based on out-of-fold predictions from the outer folds, LR_3var, XGBoost, and SVM_linear showed similar average discrimination, whereas RF yielded a lower mean outer-fold AUC (Table 4). In addition, multivariable models provided incremental discrimination compared with any single predictor alone, supporting the additive value of combining WBC, CRP, and albumin for risk estimation (Fig. 3; Supplementary Table S2).

Interpretability analyses showed consistent patterns of feature attribution across models. In XGBoost and RF, CRP contributed most strongly, followed by WBC and albumin (Supplementary Figures S4–S6), whereas in SVM_linear the relative importance ranking favored WBC, followed by CRP and albumin (Supplementary Figure S2). These convergent findings supported the relevance of inflammatory burden (WBC/CRP) and albumin-related risk stratification for gangrenous perforation among elderly patients [40].

In addition to discrimination, calibration was assessed using out-of-fold predictions. Overall agreement between predicted and observed risks was acceptable (Fig. 5), and the Brier score supported reasonable probabilistic accuracy for LR_3var (Table 4). Decision curve analysis suggested potential net benefit of multivariable models across clinically relevant threshold probabilities compared with treat-all and treat-none strategies (Fig. 6), indicating potential utility for assisting preoperative risk stratification rather than replacing clinical judgment [41].

For clinical presentation, LR_3var was selected given its interpretability and stable performance under resampling. The final LR_3var model was refitted using the full dataset to construct a nomogram as a clinical tool (Fig. 7); this refitting step was performed for presentation purposes, whereas performance estimation relied on repeated nested cross-validation. The resulting tool may facilitate rapid, noninvasive risk estimation using routinely obtained laboratory tests, particularly in settings where immediate access to advanced imaging or specialist evaluation is limited [11].

Several limitations should be acknowledged. First, this was a single-center retrospective study with a modest sample size, which may limit transportability to other institutions or healthcare systems. Second, although repeated nested cross-validation was used to reduce optimism and improve internal validity, external validation in independent cohorts remains necessary before clinical deployment [29]. Future studies should validate the model in multicenter prospective cohorts and explore whether incorporating additional readily available features can further improve performance without compromising interpretability and feasibility.

## Conclusion

A diagnostic prediction approach for gangrenous perforation in elderly patients with acute appendicitis was developed using routinely available laboratory variables. Following LASSO-based feature reduction and subsequent logistic regression screening, three predictors (WBC, CRP, and albumin) were retained for model development. Under repeated nested cross-validation, multivariable models showed improved discrimination compared with single predictors, with LR_3var, XGBoost, and SVM_linear demonstrating comparable performance and RF showing lower average discrimination. Calibration and decision curve analyses based on out-of-fold predictions supported the potential clinical usefulness of this three-variable model framework for preoperative risk stratification. For clinical implementation, a nomogram based on LR_3var was constructed to provide an interpretable tool for individualized risk estimation using routinely measured laboratory tests.

## Supplementary Information

Supplementary Material 1: Figure S1. SVM kernel comparison.Figure S2. SVM_linear feature importance.Figure S3. SVM_linear 3D decision boundary.Figure S4. XGBoost SHAP summary plot.Figure S5. XGBoost feature importance.Figure S6. RF feature importance.

Supplementary Material 2: Table S1. Univariable and multivariable logistic regression for the nine LASSO-selected predictors.Table S2. Pairwise post-hoc comparisons after the Friedman test (Nemenyi procedure).Table S3. Hyperparameter tuning strategy and final model parameters.Table S4. Nested cross-validation design and preprocessing pipeline.

## Data Availability

The datasets generated and/or analyzed during the current study are available from the corresponding author on reasonable request.

## Abbreviations

AUC: Area under the receiver operating characteristic curve
BMI: Body mass index
CI: Confidence interval
CRP: C-reactive protein
CT: Computed tomography
CV: Cross-validation
DCA: Decision curve analysis
DBIL: Direct bilirubin
df: Degrees of freedom
HCT: Hematocrit
HGB: Hemoglobin
IBIL: Indirect bilirubin
LASSO: Least absolute shrinkage and selection operator
LR_3var: Three-variable logistic regression model (using WBC, CRP, and albumin)
ML: Machine learning
MRI: Magnetic resonance imaging
Na⁺: Serum sodium
OOF: Out-of-fold
OR: Odds ratio
PCT: Procalcitonin
PLT: Platelet count
RDW: Red blood cell distribution width
RF: Random forest
ROC: Receiver operating characteristic
SVM: Support vector machine
WBC: White blood cell count
XGBoost: Extreme gradient boosting
λmin: Lambda at minimum cross-validated error
λ1se: Largest lambda within one standard error of the minimum
