# Supplementary figures and images for: Machine learning constructs a diagnostic prediction model for gangrenous perforation of acute appendicitis in elderly patients
Source: BMC Surg. 2026 Apr 24;26:387. doi: 10.1186/s12893-026-03753-y (PMC13250998; doi:10.1186/s12893-026-03753-y)

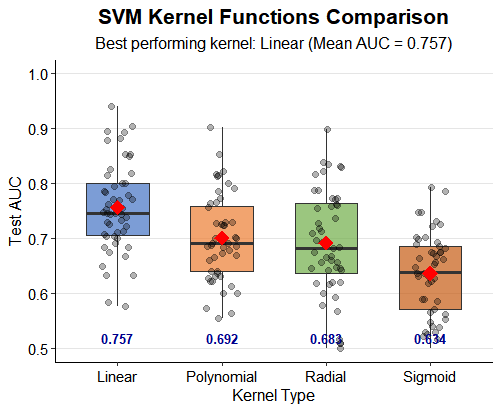


**Figure S1.**


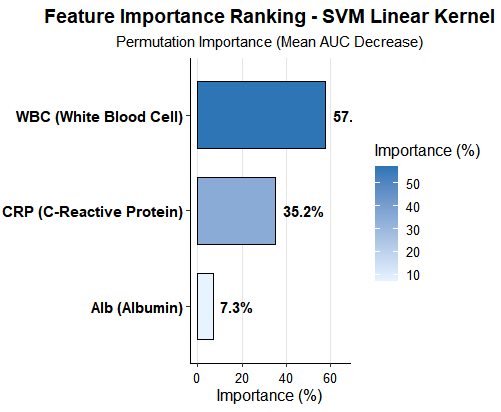


**Figure S2.**


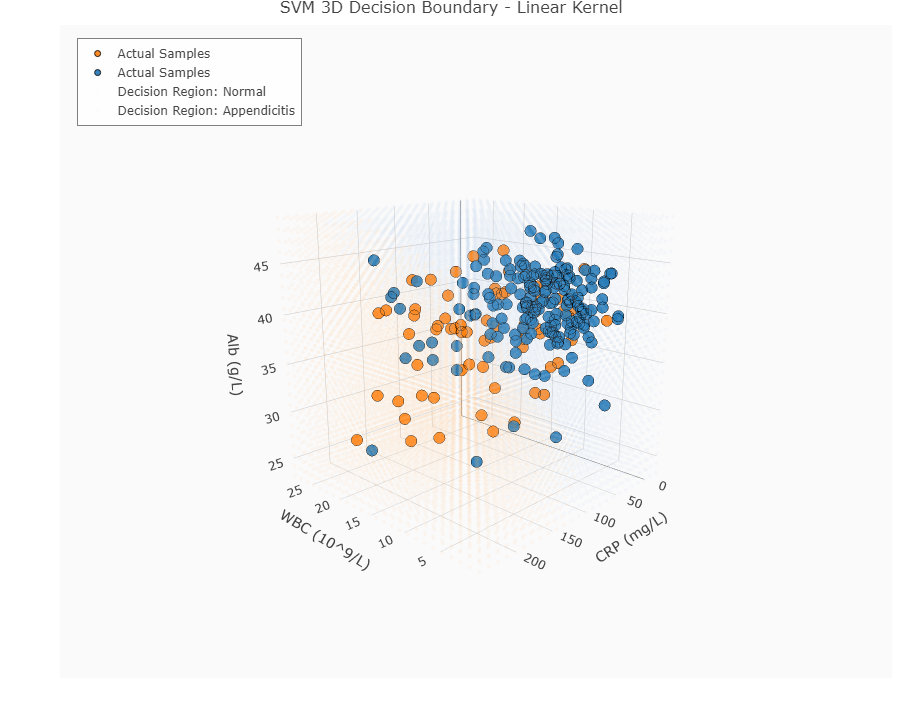


**Figure S3.**


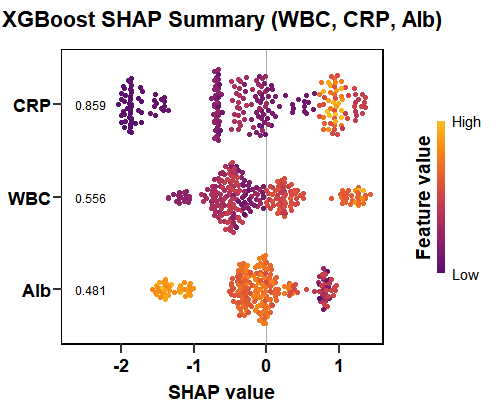


**Figure S4.**


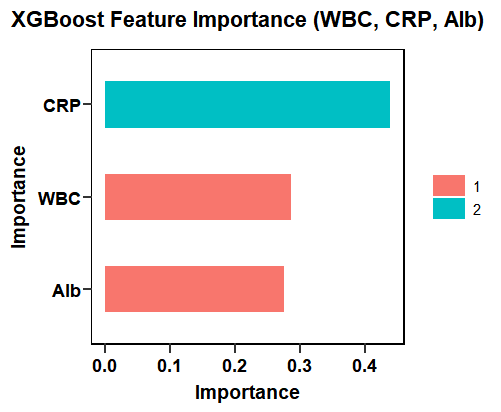


**Figure S5.**


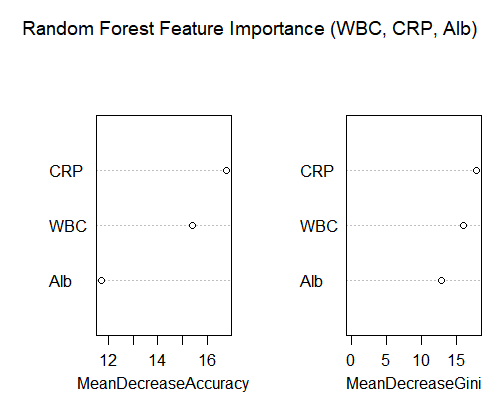


**Figure S6.**

Supplement: Supplementary file 1 — Supplementary Material 1: Figure S1. SVM kernel comparison.Figure S2. SVM_linear feature importance.Figure S3. SVM_linear 3D decision boundary.Figure S4. XGBoost SHAP summary plot.Figure S5. XGBoost feature importance.Figure S6. RF feature importance. [file 12893_2026_3753_MOESM1_ESM.docx]
