# Supplementary material for: Machine learning constructs a diagnostic prediction model for gangrenous perforation of acute appendicitis in elderly patients
Source: BMC Surg. 2026 Apr 24;26:387. doi: 10.1186/s12893-026-03753-y (PMC13250998; doi:10.1186/s12893-026-03753-y)
Supplement: Supplementary file 2 — Supplementary Material 2: Table S1. Univariable and multivariable logistic regression for the nine LASSO-selected predictors.Table S2. Pairwise post-hoc comparisons after the Friedman test (Nemenyi procedure).Table S3. Hyperparameter tuning strategy and final model parameters.Table S4. Nested cross-validation design and preprocessing pipeline. [file 12893_2026_3753_MOESM2_ESM.docx]

| **Supplementary Table S1. Univariable and multivariable logistic regression for the nine LASSO-selected predictors.** | | | | |
| --- | --- | --- | --- | --- |
| **Characteristics** | **Single factors LR** | | **Multiple factors LR** | |
|  | ***OR* (95%CI*)*** | ***P*-value** | ***OR* (95%CI*)*** | ***P*-vlaue** |
| **Smoking** | 3.45 (1.41-8.47) | 0.007 | 2.72 (0.90-8.20) | 0.076 |
| **Fever** | 4.33 (1.93-9.71) | <0.001 | 2.03 (0.74-5.51) | 0.167 |
| **WBC*** | 1.16 (1.06-1.26) | <0.001 | 1.12 (1.01-1.25) | 0.038 |
| **LYM%** | 0.91 (0.86-0.97) | 0.002 | 0.95 (0.88-1.03) | 0.221 |
| **PCT** | 1.04 (1.01-1.08) | 0.007 | 1.01 (0.97-1.05) | 0.640 |
| **CRP*** | 1.01 (1.01-1.02) | <0.001 | 1.01 (1.00-1.01) | 0.041 |
| **Alb*** | 0.84 (0.76-0.92) | <0.001 | 0.85 (0.76-0.95) | 0.004 |
| **Na+** | 0.87 (0.78-0.97) | 0.011 | 0.94 (0.85-1.03) | 0.194 |
| **Appendiceal wall thickening** | - | 0.986 | - | - |
| **Note**: *Variables retained in the final 3-variable multivariable logistic regression model. | | | | |

| **Supplementary Table S2. Pairwise post-hoc comparisons after the Friedman test (Nemenyi procedure).** | | | |
| --- | --- | --- | --- |
| **S2A. Overall Friedman test** | | | |
| **Test** | **Statistic** | ***df*** | ***P*-value** |
| **Friedman rank-sum test** | 104.28 | 6 | < 2.2 × 10^-16^ |
| **S2B. Pairwise post-hoc comparisons (Nemenyi test)** | | | |
| **Comparison** |  | **Adjusted *P*-value** |  |
| **Random forest vs LR_3var** |  | 0.09060 |  |
| **SVM_linear vs LR_3var** |  | 0.99741 |  |
| **XGBoost vs LR_3var** |  | 1.00000 |  |
| **LR_WBC vs LR_3var** |  | < 0.001 |  |
| **LR_CRP vs LR_3var** |  | 0.0358 |  |
| **LR_Alb vs LR_3var** |  | < 0.001 |  |
| **SVM_linear vs Random forest** |  | 0.25079 |  |
| **XGBoost vs Random forest** |  | 0.01392 |  |
| **LR_WBC vs Random forest** |  | < 0.001 |  |
| **LR_CRP vs Random forest** |  | < 0.001 |  |
| **LR_Alb vs Random forest** |  | < 0.001 |  |
| **XGBoost vs SVM_linear** |  | 1.00000 |  |
| **LR_WBC vs SVM_linear** |  | < 0.001 |  |
| **LR_CRP vs SVM_linear** |  | < 0.001 |  |
| **LR_Alb vs SVM_linear** |  | < 0.001 |  |
| **LR_WBC vs XGBoost** |  | < 0.001 |  |
| **LR_CRP vs XGBoost** |  | < 0.001 |  |
| **LR_Alb vs XGBoost** |  | < 0.001 |  |
| **LR_CRP vs LR_WBC** |  | 1.00000 |  |
| **LR_Alb vs LR_WBC** |  | 0.99998 |  |
| **LR_Alb vs LR_CRP** |  | 1.00000 |  |
| Note: Post-hoc comparisons were performed using the Nemenyi procedure following the Friedman rank-sum test. | | | |

| **Supplementary Table S3. Hyperparameter tuning strategy and final model parameters.** | | | |
| --- | --- | --- | --- |
| **Model** | **Tuning strategy** | **Hyperparameter search space** | **Final selected parameters** |
| **LR_3var** | Repeated nested CV with 5 outer folds, 5 inner folds, and 10 repeats; no hyperparameter tuning | None | Standard multivariable logistic regression with WBC, CRP, and Alb |
| **XGBoost** | Repeated nested CV; random search within the predefined grid in each inner-loop training set; model selection based on inner CV AUC | max_depth = {2, 3, 4}; eta = {0.01, 0.05, 0.1}; gamma = {0.1, 0.5}; subsample = 0.8; colsample_bytree = 0.8; min_child_weight = {3, 5}; lambda = {1, 5}; alpha = {0, 0.1}; nrounds = 200 with early stopping (15 rounds) | max_depth = 2; eta = 0.1; gamma = 0.5; subsample = 0.8; colsample_bytree = 0.8; min_child_weight = 5; lambda = 1; alpha = 0; scale_pos_weight = 2.637681 |
| **RF** | Repeated nested CV; random search within the predefined grid in each inner-loop training set; model selection based on inner CV AUC | ntree = {100, 300, 500}; mtry = {1, 2, 3}; nodesize = {5, 10, 20} | ntree = 500; mtry = 1; nodesize = 20 |
| **SVM** | Repeated nested CV; full grid search for each kernel in inner-loop training; best kernel selected according to mean AUC | **Linear**: *C* = {0.01, 0.1, 1, 10, 100}; **Polynomial**: *C* = {0.01, 0.1, 1, 10, 100}, degree = {2, 3, 4}, scale = {0.01, 0.1, 1}; **Radial**: *C* = {0.01, 0.1, 1, 10, 100}, gamma = {0.001, 0.01, 0.1, 1}; **Sigmoid**: *C* = {0.01, 0.1, 1, 10, 100}, gamma = {0.001, 0.01, 0.1, 1} | Best kernel: linear; final parameter: *C* = 10 |
| Abbreviations: CV, cross-validation; AUC, area under the receiver operating characteristic curve. | | | |

| **Supplementary Table S4. Nested cross-validation design and preprocessing pipeline.** | |
| --- | --- |
| **Item** | **Setting / Description** |
| Outcome variable | Diagnosis (negative vs positive) |
| Candidate predictors used in final models | WBC, CRP, Alb |
| Outer cross-validation | 5-fold |
| Inner cross-validation | 5-fold |
| Number of repeats | 10 |
| Random seed | 123 |
| Model selection principle | Best-performing hyperparameters selected within inner-loop CV according to AUC |
| Missing data handling | Complete-case analysis (no imputation); only patients with complete perioperative data were included. |
| Standardization | Continuous predictors were centered and scaled using training-set statistics before SVM fitting; fold-specific scaling parameters were applied to the corresponding test fold |
| Threshold selection for classification | Optimal threshold determined by the Youden index within the ROC analysis of each outer test fold |
| Leakage control | Standardization was performed within each resampling split using training data only; fold-specific scaling parameters were applied to the corresponding test fold |
| Final model fitting | After model comparison, the final selected model was refit using the full dataset |
